# Eco-friendly synthesis of Carbon Quantum Dots (CQDs) from hazelnut husk for sensitive Aflatoxin B1 (AFB1) detection

**DOI:** 10.1016/j.toxrep.2024.101824

**Published:** 2024-11-22

**Authors:** Hatice Yuncu, Hayrunnisa Nadaroglu, Ebru Bozkurt

**Affiliations:** a Department of Nano‑Science and Nano‑Engineering, Institute of Science, Ataturk University, Erzurum 25240, Turkey; b Department of Food Technology, Vocational College of Technical Sciences, Ataturk University, Erzurum 25240, Turkey; c Department of Occupational Health and Safety, Vocational College of Technical Sciences, Ataturk University, Erzurum 25240, Turkey

**Keywords:** Hazelnut husk waste, Carbon Quantum Dots (CQDs), Aflatoxin B1 (AFB1), Fluorescent probe

## Abstract

In this study, green fluorescent carbon quantum dots (CQDs) with remarkable stability, water solubility, and biocompatibility were synthesized from hazelnut husk (HH) waste material using a novel approach by the pyrolysis method. The optical properties of the synthesized HH-CQDs were characterized by UV-Vis and fluorescence spectroscopy (PL), while their structural properties were characterized using various techniques, including transmission electron microscopy (TEM), Fourier transform infrared spectroscopy (FT-IR), X-ray diffraction (XRD), and X-ray photoelectron spectroscopy (XPS). TEM images revealed that HH-CQDs had a spherical shape with diameters ranging from 2 to 10 nm. The fluorescence quantum yield of these CQDs was measured as 0.04. Furthermore, CQDs were very effective at finding aflatoxin B1 (AFB1) using a fluorescence resonance energy transfer (FRET) mechanism, with a clear fluorescence emission peak seen at 451 nm. The photoluminescent properties of CQDs were evaluated under various pH conditions, showing a blue shift and increased fluorescence intensity at pH 9–10, suggesting their potential use in pH-sensitive sensor applications. This study demonstrates the selective and sensitive detection of AFB1 using HH-CQDs, with a strong linear relationship (R² = 0.9936) between fluorescence intensity and AFB1 concentration in the range of 25–250 ppm, and high accuracy in real food samples, including 81.56 % in corn, 98.64 % in milk, and 95.73 % in peanuts. This eco-friendly and cost-effective synthesis method offers a promising alternative for AFB1 detection in food samples by utilizing waste material to create valuable analytical tools.

## Highlights

• Green fluorescent CQDs from hazelnut husk waste via pyrolysis exhibited stability, water solubility, and biocompatibility.
• CQDs (2–10 nm) analyzed via UV-Vis, PL, TEM, FT-IR, XRD, and XPS showed spherical structure with a fluorescence quantum yield of 0.04.
• CQDs detected aflatoxin B1 with enhanced sensitivity at pH 9–10, highlighting potential use as pH-sensitive food safety sensors.

## Graphical Abstract

**Figure:**
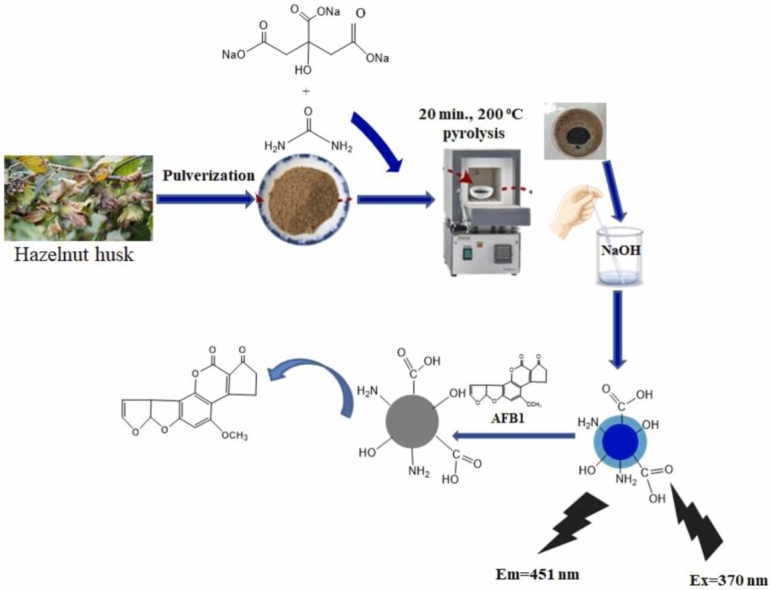

## 1 Introduction

The contamination of agricultural crops with molds before or after harvest, including those caused by improper storage, poses a significant health threat to consumers. The subsequent processing of raw materials often fails to remove the compounds they secrete, particularly mycotoxins, which are resistant to treatment [1]. One of the most common and persistent toxins is aflatoxins, including aflatoxin B1 (AFB1), which is classified as a class 1 carcinogen by the International Agency for Research on Cancer. Agricultural products like maize, corn, and nuts are most susceptible to AFB1 contamination, with the European Commission setting maximum residue limits of 2 µg/kg in cereals and grain-containing products [2]. The problem of food contamination with AFB1 is of significant economic importance due to its threat to human and livestock health and the marketing of agricultural products [3], [4].

AFB1 detection methods include liquid chromatography-mass spectrometry, gas chromatography-tandem mass spectrometry, enzyme-linked immunosorbent assay (ELISA), and immunochromatographic assay [5]. Each method has its advantages and disadvantages. Chromatographic methods offer high accuracy but are time-consuming and expensive due to complex pretreatment processes. ELISA and immunochromatography are user-friendly but require time-consuming immobilization of reagents [6]. Conventional antibodies offer good sensitivity but face issues like denaturation, batch-to-batch variability, and chemical modification [7].

The field of biosensing and environmental monitoring has experienced a surge in interest in nanotechnology, particularly in the synthesis and utilization of carbon-based nanomaterials. Carbon quantum dots (CQDs) have garnered significant attention due to their unique optical characteristics, biocompatibility, and potential for functionalization. These nanomaterials, typically less than 10 nanometers in size, exhibit exceptional fluorescence, high stability, and low toxicity, making them suitable for various applications such as bioimaging, drug delivery, and pollutant detection [8], [9], [10].

Fluorescent biosensors are gaining popularity due to their simplicity, low cost, convenience, and high sensitivity as a reliable alternative [11]. Their distinct properties have positioned them as promising candidates for a wide range of applications, including enzyme activity assays, fluorescent identification of cancer cells and bacteria, and antibacterial applications [12], [13]. The synthesis techniques, size control, surface modification approaches, optical properties, and luminescent mechanisms of CQDs have been extensively researched, underscoring their potential in bioimaging, biosensing and catalysis [14].

The versatility of CQDs extends to their applications in live cell imaging, inflammation treatment, photocatalysis, electronics, nanomedicine, chemical sensing, biosensing, photodynamic therapy, pharmaceutical formulations, targeted drug delivery, and other biomedical fields due to their unique properties [9], [15], [18]. Additionally, the peroxidase-like catalytic activities of CQDs have been explored for biosensing applications, demonstrating their potential in this area [15]. Recent advancements in the synthesis of doped CQDs from carbon-rich sources have introduced new possibilities for biomedical and sensing applications [16].

These nanomaterials have been employed in the preparation of dopamine fluorescence probes, cellular imaging, and as fluorescent nanomaterials with broad application prospects in biosensing and optoelectronics [17]. Many plants, fruits, grains, and different organic materials can be used as natural carbon (C) sources in the synthesis of carbon dots from waste. CQDs were produced quickly with low cost and ease of synthesis by the pyrolysis synthesis method. The synthesis of CQDs was used with very few changes in the method used by Kostromin et al. [18]. Moreover, the controlled emission of CQDs derived from waste silk sericin has shown potential for applications in optoelectronic devices, bioimaging, and biosensing.

In this study, we explore the synthesis of carbon quantum dots from hazelnut husk, focusing on their structural and optical characteristics. Furthermore, we investigate the application of these CQDs in the detection of aflatoxins, a group of toxic and carcinogenic compounds produced by certain molds found in food and feed. Aflatoxin contamination poses a serious threat to food safety and public health, necessitating the development of reliable and sensitive detection methods.

The objective of this research is to demonstrate the feasibility of using hazelnut husk-derived CQDs as a fluorescent probe for the detection of aflatoxins. By harnessing the unique properties of CQDs, we aim to develop a rapid, sensitive, and cost-effective detection platform that can be integrated into food safety monitoring systems. This study advances the sustainable synthesis of CQDs while addressing key challenges in food safety and environmental monitoring.

## 2 Material and methods

### 2.1 Chemicals and materials

Ammonium citrate (C_6_H_17_N_3_O_7_) (Merck), urea (CH_4_N_2_O) (Merck) and sodium hydroxide (NaOH) were purchased from Sigma—Aldrich in powder form. Aflatoxin B1 (AFB1 mg/ml) was obtained from Sigma—Aldrich in powder form. Deionized water and methanol were used as solvents in all experiments.

### 2.2 Synthesis of CQDs

Hazelnut husk (HH) waste material was sourced from Artvin, Turkey and recognized by the Biology Department of the Faculty of Science. The nuts were dried at room temperature, washed first with dishwasher water and then with pure water. The nuts were then dried and soaked with liquid nitrogen and turned into powder in the oven. The synthesis of CQDs was used with little modification of the method used by Kostromin et al. [18]. In short, 0.005 g of HH was taken and placed in a porcelain crusher, and again heavily beaten in the air to become a powder. 0.1 g of ammonium citrate and 0.05 g of urea, diluted in the oven, were then added to the oven for 25 minutes at 200 degrees. It was then cooled to room temperature in the desiccator, and the resulting black-coloured reaction content was transferred to the experimental tube, then added 4 ml of 0.25 NaOH solution and 6 ml of pure water and mixed in the shaker for 30 mins. It was then centrifugated at +4 *°*C for 30 mins at 10,000 xg. Finally, to remove unreacted chemicals, all synthesized Cu NCs were dialyzed against distilled water for 24 hours at room temperature. The final product was stored in amber-coloured bottles at *+*4 *°*C until study [19]. Here, when preparing CQDs, the reproducibility of the synthesis is quite high in terms of the presence of abundant waste products, optimization of synthesis conditions, and adjustment of pH and temperature.

**Fig. 1:**
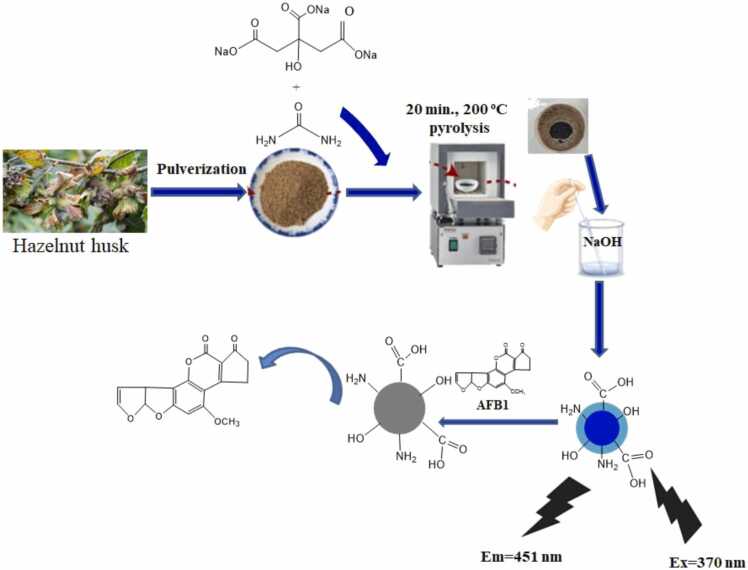
Schematic scheme for the detection of aflatoxin B1 (AFB1) by hydrothermal assisted synthesis of HH-CQDs through the Floresan Resonance Energy Transfer Mechanism (FRET).

After the Hazelnut husk CQD (HH-CQDs) was prepared, the optical properties of the HH-CQDs were distinguished by UV–vis and florasan spectroscopy techniques. The quantum yield of the obtained HH-CQDs (*%*QY) was calculated using the integrated emission density of kin sulfate as standard. The flora distribution was tested at different times under light to investigate the photostability of the prepared HH-CQDs. All routine procedures are shown schematically in Fig. 1.

### 2.3 Fluorescence intensity determination of AFB1

The impact of Aflatoxins B1 (AFB1) on the fluorescence intensity of HH-CQDs was assessed by introducing varying concentrations of AFB1 (ranging from 0.25 to 2 ng/ml) to HH-CQDs solutions (20 µg/ml). The mixtures were incubated at room temperature for periods ranging from 30 minutes to 1 hour, after which the fluorescence intensities were recorded at various excitation/emission wavelengths. Moreover, experimental conditions such as pH, temperature, and incubation time were optimized for the detection of AFB1. The calibration curve for AFB1 was established based on the alterations observed in the fluorescence spectra of the HH-CQDs [19].

### 2.4 Detection of AFB1 in real samples using HH-CQD

AFB1 was detected in real samples (milk, corn, and peanut) using HH-CQDs prepared under optimum conditions. All experiments were performed on milk, corn obtained from local markets in Erzurum, Turkey, and peanuts obtained from Osmaniye, Turkey. 8 g of corn and peanuts were taken and crushed, 40 ml of pure water + alcohol (8 ml of alcohol: 2 ml of pure water) was added, and blended. It was kept for half an hour. It was filtered and centrifuged for 20 minutes. The sediment was discarded, and the supernatant was taken. It was kept at +4 C° until used. The milk sample (5 ml) was centrifuged at 9000 rpm for 20 minutes, and the fat part was taken. A mixture of 10 ml of pure water and alcohol (8:2) was prepared, and 5 ml of the alcohol and water mixture was added to 1 ml of milk. HH-CQD was added to real samples to which AFB1 was added at known concentrations, and changes were measured by fluorescence intensity measurements.

## 3 Results

### 3.1 Characterization of the synthesized HH-CQDs

To create a nanobiosensor for detecting and monitoring AFB1 in food samples through sequential fluorescence switching in a single aqueous system, urea served as both the ligand and reducing agent. This resulted in the production of fluorescent HH-CQDs with -NH groups that offer excellent stability, water solubility, and biocompatibility. These HH-CQDs were synthesized using a rapid, environmentally friendly method (Fig. 1).

### 3.2 Quantum efficiency measurement

The quantum yield (QY) of the HH-CQDs was determined by comparing their photoluminescence and absorbance values to those of quinine sulfate. Various concentrations of the solutions were prepared, ensuring the absorbance of the HH-CQDs solution remained below 0.10 at an excitation wavelength of 360 nm. Quinine sulfate, with a known QY of 0.54, was dissolved in 1 mmol/L H_2_SO_4_ and used as the reference standard for the integrated density measurements.

The quantum efficiency was calculated using the following equation:

$$Q_{X}=Q_{R}\left[\frac{I_{X}}{A_{X}}\right]\left[\frac{A_{R}}{I_{R}}\right]{\left[\frac{n_{X}}{n_{R}}\right]}^{2}$$

• I is the measured integrated emission intensity,
• A, optical density,
• n is the refractive index of the solvent, and
• Subscript X represents the fluorophore of the sample,
• Subscript R denotes the fluorophore of the standard.

In this study, the QY of HH-CQDs was calculated to be 0.04

The HH-CQDs synthesized via the pisolysis method were characterized using UV–vis absorption, fluorescence spectroscopy, TEM, FT-IR, XRD and XPS techniques.

**Fig. 2:**
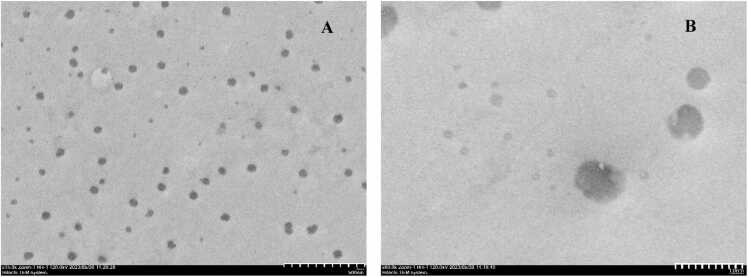
TEM analysis (A and B) of HH-CQDs.

Transmission electron microscopy (TEM) provided detailed information on the size and morphology of the HH-CQDs, confirming their formation as depicted in Fig. 2A. The HH-CQDs exhibited a uniform, spherical morphology with an average particle size ranging from 2 to 10 nm (Fig. 2A and B).

TEM experiments were conducted to examine the morphology and dimensions of HH-CQDs. As illustrated in Fig. 1A, the HH-CQDs were uniformly distributed and consistently sized. The particle sizes ranged from approximately 1.28 to 3.10 nm, with an average size of 2.08 nm, as depicted in the accompanying histogram. High-resolution TEM (HRTEM) images revealed an interlayer lattice spacing of 0.23 nm, corresponding to the diffraction planes of graphitic carbon [20].

### 3.3 FTIR analysis

**Fig. 3:**
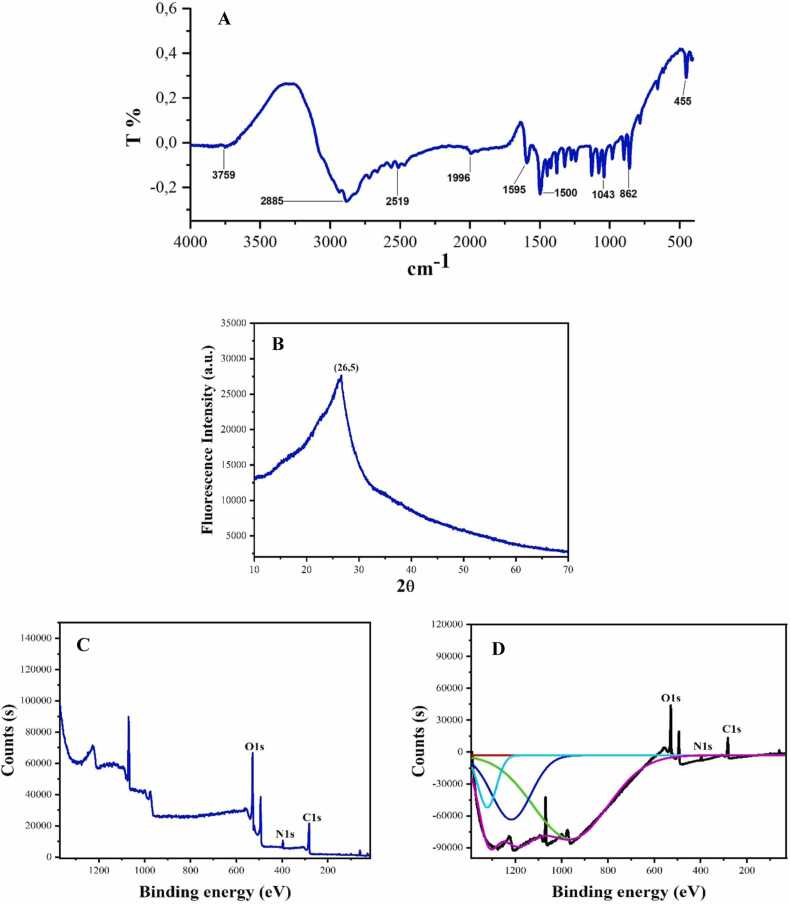
FTIR analysis (A), XRD patterns (B), XPS spectra (C) and survey XPS spectrum (D) of HH-CQDs.

The Fourıer transform ınfrared spectrometer (FTIR) of HH-CQDs is presented in Fig. 3A. Stretching vibrations for –OH and –NH_2_ groups (3300–3000 *cm*^*−1*^), C–H (2885 *cm*^*−1*^ and 2519 *cm*^*−1*^), C=O (1595 *cm*^*−1*^), C=C (1500 *cm*^*−1*^), C–O–C (1340 *cm*^*−1*^), C–N (1188 *cm*^*−1*^) and C–O (1043 *cm*^*−1*^) reveal the presence of conjugated aromatic structure consisting of N- and O- containing [21], [22], [23].

The functional groups present on the surface of HH-CQDs enhance their ability to interact with aflatoxin molecules. Hydroxyl and amine groups can form hydrogen bonds with aflatoxin, while carbonyl and ether groups engage in other non-covalent interactions. These specific interactions contribute to the high sensitivity and selectivity of the HH-CQDs, facilitating the effective capture and recognition of aflatoxin molecules and enabling accurate and reliable detection.

In comparison to existing studies, the FTIR spectrum of HH-CQDs exhibits similar functional group patterns, highlighting the successful incorporation of hydroxyl and amine groups that are essential for the interaction with target molecules like aflatoxin. The carbonyl and ether groups present in the HH-CQDs are also observed in other studies involving CQDs, where they contribute to non-covalent interactions such as hydrogen bonding and π-π stacking with various analytes. These interactions are crucial for the high sensitivity and selectivity of HH-CQDs in detecting aflatoxin. The presence of these functional groups in HH-CQDs supports their ability to form hydrogen bonds with aflatoxin molecules, enhancing their capability for accurate and reliable detection, a characteristic similar to that found in other CQD-based sensors.

### 3.4 XRD analysis

The crystal structures of HH-CQDs were analyzed using X-ray diffraction (XRD). As depicted in Fig. 3B, the XRD spectrum of the CQDs displayed a broad peak centered around 26.5*°*, corresponding to the (002) planes of graphite. This observation is indicative of a graphitic structure, suggesting the presence of sp²-hybridized carbon atoms arranged in a layered configuration. The broad peak at 26.5*°* aligns with previously reported findings, confirming the formation of graphite-like carbon quantum dots [21], [24]. The presence of this peak suggests that the HH-CQDs have retained a crystalline structure, which is essential for their electronic and optical properties. The graphitic nature of these CQDs enhances their electrical conductivity and stability, making them suitable for sensing AFB1.

### 3.5 XPS analysis

The composition of HH-CQDs was quantitatively analyzed using X-ray photoelectron spectroscopy (XPS) (Specflex VersaProbe). The XPS survey spectra, displayed in Fig. 3C, reveal three prominent peaks corresponding to C 1 s at 285.06 eV, N 1 s at 400.01 eV, and O 1 s at 531.85 eV. These peaks confirm the presence of carbon, nitrogen, and oxygen in the HH-CQDs.

The C 1 s peak at 285.06 eV indicates various carbon bonding environments, such as sp2 and sp3 hybridizations, suggesting a complex carbon network in the HH-CQDs. This complexity is crucial for the photoluminescent properties of the carbon dots, which are essential for detecting aflatoxins. The N 1 s peak at 400.01 eV confirms the successful doping of nitrogen atoms into the carbon dots. Nitrogen doping enhances the electronic properties of carbon dots, improving their photoluminescence and making them more suitable for sensitive and selective detection of aflatoxins. Nitrogen atoms introduce additional electronic states, which can interact with AFB1 molecules, leading to detectable changes in fluorescence. The O 1 s peak at 531.85 eV signifies the presence of oxygen-containing functional groups, such as hydroxyl, carbonyl, and carboxyl groups, on the surface of the HH-CQDs. These functional groups enhance the hydrophilicity and stability of the carbon dots in aqueous solutions, which is crucial for practical applications in aflatoxin detection. The oxygen groups also facilitate specific interactions with aflatoxin molecules, improving the selectivity of the detection system. In the context of aflatoxin detection, the photoluminescent properties of HH-CQDs can be exploited to develop sensitive and selective sensors. When exposed to aflatoxin molecules, the fluorescence of the HH-CQDs can change, providing a measurable signal that indicates the presence and concentration of aflatoxins. The combination of nitrogen doping and oxygen-containing functional groups enhances the interaction between the HH-CQDs and aflatoxins, leading to improved sensitivity and selectivity of the detection system [25], [26], [27]. From XPS elemental analysis, CQDs were determined to have a composition of 50.9 % C, 42.9 % O and 6.1 % N (Fig. 3).

### 3.6 Fluorescence and absorbance analysis results

**Fig. 4:**
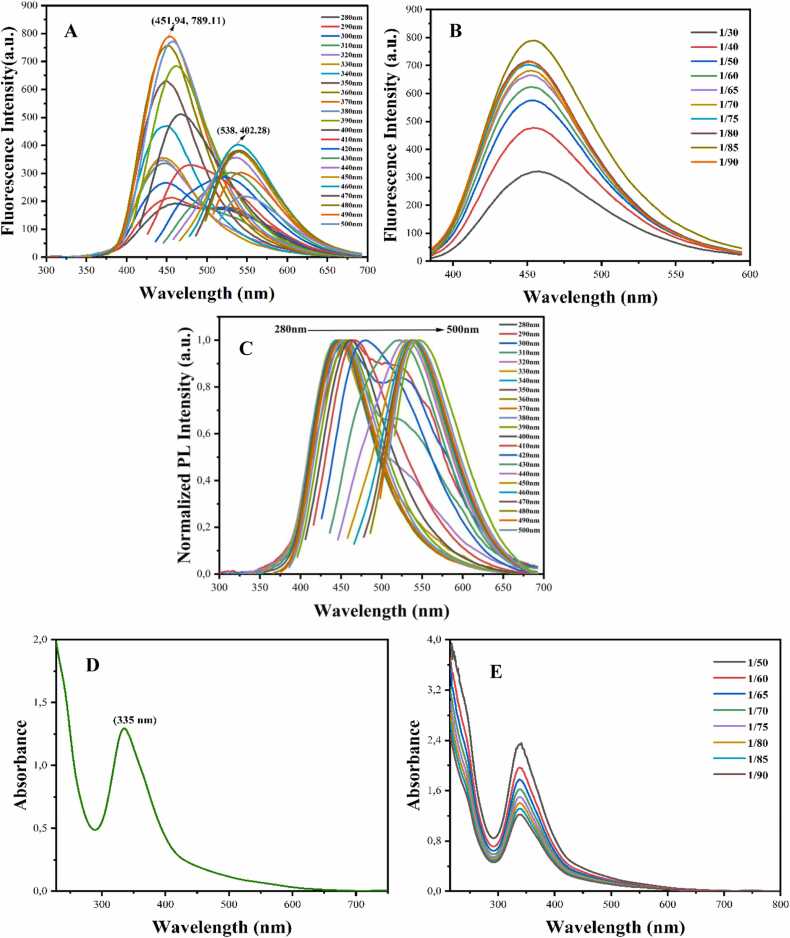
Fluorescence spectra of HH-CQDs (A), fluorescence spectra of diluted HH-CQDs (B), normalized fluorescence spectra (C), absorbance spectra of HH-CQDs at various excitation wavelengths ranging from 280 nm to 400 nm with 10 nm increments (D and E).

Excitation fluorescence and absorption measurements were taken for the optical characterization of the obtained HH-CQDs. Fluorescence measurements were also taken to determine the fluorescence character of the synthesized HH-CQDs structure. A strong emission peak at 451 nm when excited at 370 nm proved that HH-CQDs possess fluorescence properties (Fig. 4A). The dilution of HH-CQDs increased the fluorescence intensity, with the highest dilution of 1/85 yielding the strongest peak (Fig. 4B). It was investigated the dependence of HH-CQDs fluorescence intensity on the excitation wavelength by varying the excitation wavelength between 280 and 400 nm, and Fig. 4C presents the results with the normalized fluorescence spectrum. It was observed that the fluorescence intensity of HH-CQDs decreased, and the luminescence peak gradually shifted from 440 nm to 462 nm with increasing excitation wavelength. For all measurements, we chose an excitation wavelength of 370 nm to achieve the maximum fluorescence of HH-CQDs at 451 nm. The 334 nm peak is made up of different electronic transitions, such as π-π*, n-π*, and π. These happen because of heteroatoms like nitrogen (C=N), amines (C-NH_2_), and aromatic carbon bonds (C=C). It is observed due to -π*). These electronic passes are: the σ-σ* transition that occurs between the energy levels of two sigma (σ) bonds; the n-σ* transition, which occurs between the free electron pairs (n) in the molecule and the anti-bonding orbitals (σ*) of sigma bonds; and the pi (π) transition. It refers to the π-π* transition that occurs between the energy levels of bonds.

A sharp peak around 335 nm was observed in the UV-Vis absorption spectrum of HH-CQDs (Fig. 4). The UV-Vis absorption spectrum of HH-CQDs exhibited a sharp peak around 335 nm, indicative of the characteristic optical properties of carbon quantum dots (CQDs). This absorption feature can be attributed to the π-π* transitions of the aromatic C=C bonds within the carbon core structure of the CQDs. The presence of such a peak is consistent with previous studies on CQDs, which often report similar absorption maxima due to their unique electronic properties and quantum confinement effects.

Comparatively, the absorption spectra of CQDs can vary based on factors such as the synthesis method, surface functionalization, and the presence of heteroatoms or functional groups. For instance, Sun et al. [28] demonstrated that CQDs synthesized via different routes exhibit absorption peaks ranging from the UV to the visible region, influenced by their surface chemistry and core structure. Similarly, Zhou et al. [29] reported that nitrogen-doped CQDs showed absorption features that shifted depending on the extent of doping and the nature of the surface groups. In our study, the sharp absorption peak around 335 nm underscores the effectiveness of the synthesis method employed for producing HH-CQDs with desirable optical properties. Such well-defined absorption characteristics are indicative of the high quality and purity of the CQDs, making them suitable candidates for various applications that rely on their optical responses.

**Fig. 5:**
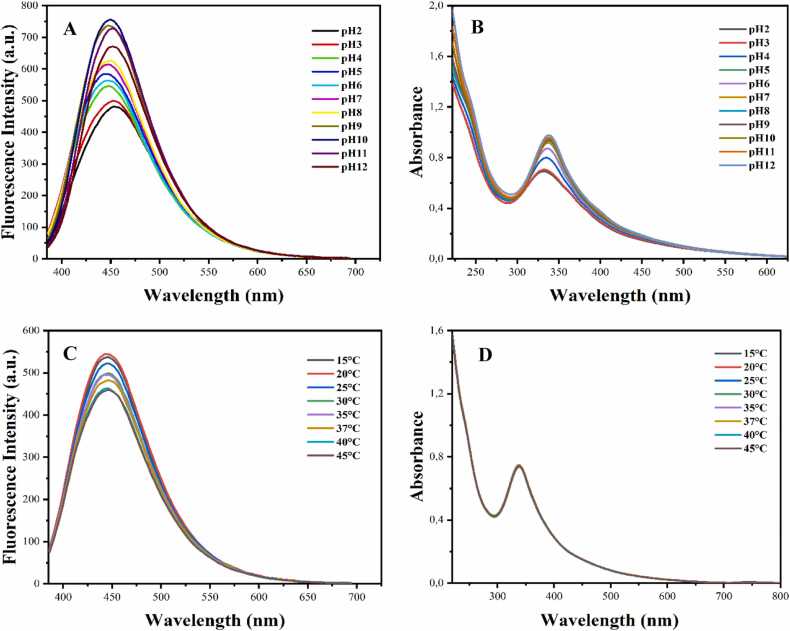
PL spectra (A) and UV-Vis spectra (B) at different pH values (pH 1–12), PL spectra (C) and UV-Vis spectra (D) at different temperatures (15–45 *°*C) of HH-CQDs (λexc = 370 nm).

The effect of pHs on the optical properties of the obtained HH-CQDs was examined and changes in the absorption and fluorescence spectrum of HH-CQDs depending on pH between pH: 1–12 are given in Fig. 5. From the findings, it was observed that at pH: 9 and 10, the HH-CQDs fluorescence intensity increased and the peak maximum shifted to blue, while in extremely acidic environments (pH: 2 and 3), the HH-CQDs fluorescence intensity decreased and the peak maximum shifted to red (Fig. 5 A).

At pH levels of 9 and 10, we observed an increase in the fluorescence intensity of the HH-CQDs, accompanied by a blue shift in the peak maximum. This enhancement in fluorescence and the shift towards shorter wavelengths suggest that the HH-CQDs exhibit improved quantum yield and reduced non-radiative recombination processes under mildly basic conditions. The blue shift is indicative of a more compact electronic structure, likely due to deprotonation of surface functional groups, which reduces electron-donating effects and results in higher energy transitions.

These pH-dependent optical properties of HH-CQDs highlight their sensitivity to the protonation state of their surface functional groups. Such sensitivity can be leveraged in designing pH-sensitive sensors and bioimaging agents, where the optical response of HH-CQDs can serve as a direct indicator of the local pH environment. Previous studies have reported similar pH-responsive behavior in CQDs, further supporting our observations. For instance, Zhao et al. [31] demonstrated that the fluorescence of CQDs synthesized from different carbon sources showed significant changes in intensity and wavelength with varying pH, which were ascribed to the protonation and deprotonation of surface functional groups. Moreover, Stefanakis et al. [32] reported that nitrogen-doped CQDs exhibited enhanced fluorescence and a blue shift under basic conditions, attributed to the deprotonation of amine groups on the CQD surface. These parallels suggest that the pH-dependent optical behavior observed in HH-CQDs is a general characteristic of CQDs, governed by their surface chemistry and environmental interactions.

The optical properties of HH-CQDs were further examined by evaluating their UV-Vis absorption spectra across a wide pH range (pH 2–12). As illustrated in Fig. 5B, there is a clear trend in the peak absorbance values and the corresponding wavelengths with changing pH levels. Specifically, the maximum absorbance increased from 0.6 at pH 2–1.1 at pH 12, accompanied by a red shift in the wavelength from 335 nm to 350 nm (Fig. 5B). The observed increase in peak absorbance with rising pH suggests that the optical density of the HH-CQDs is enhanced in basic environments. This enhancement could be due to the deprotonation of surface functional groups, such as carboxyl and hydroxyl groups, which results in increased electronic interactions and improved light absorption. The increase in absorbance can also be attributed to the improved dispersion and reduced aggregation of CQDs in basic conditions, which facilitates greater interaction with incident light. Such behavior is consistent with the pH-dependent optical properties reported for other types of CQDs. For example, Kong et al. found that the absorption and fluorescence properties of CQDs varied significantly with pH, which was attributed to changes in the surface chemistry and electronic structure [30].

The effect of temperature on the optical properties of the obtained HH-CQDs was examined. The absorption and fluorescence spectra obtained for HH-CQDs depending on the temperature in the range of 15–45 *°*C are given in Fig. 5 C and D. The findings indicate a pronounced temperature-dependent behavior in the fluorescence intensity of HH-CQDs, while their absorbance remained unchanged. The fluorescence intensity of HH-CQDs reached its peak at 20*°*C, suggesting that this temperature provides optimal conditions for their electronic transitions, resulting in the highest quantum yield. This peak in fluorescence intensity at 20*°*C can be attributed to the balance between radiative and non-radiative recombination processes within the CQDs. At this temperature, non-radiative processes such as phonon interactions are minimized, leading to maximum fluorescence efficiency. This behavior aligns with the understanding that lower temperatures generally reduce non-radiative decay pathways, allowing for higher fluorescence outputs. As the temperature increased beyond 20*°*C, a gradual decrease in fluorescence intensity was observed. This decline is likely due to the increased thermal energy, which enhances non-radiative recombination processes, such as phonon interactions and collisional quenching. These processes compete with radiative recombination, resulting in reduced fluorescence intensity at higher temperatures. The decrease in fluorescence with rising temperature is a well-documented phenomenon in quantum dots and other fluorescent nanomaterials. For instance, Wen et al. reported a similar temperature-dependent decrease in the fluorescence intensity of silicon quantum dots, attributing it to the increased non-radiative relaxation pathways at elevated temperatures [31].

### 3.7 HH-CQD selectivity for AFB1 detection

AFB1 is considered a highly potent carcinogenic compound. In this study, we synthesized HH-CQDs with bright blue fluorescence using a single-step, environmentally friendly, and economical pyrolysis method.

**Fig. 6:**
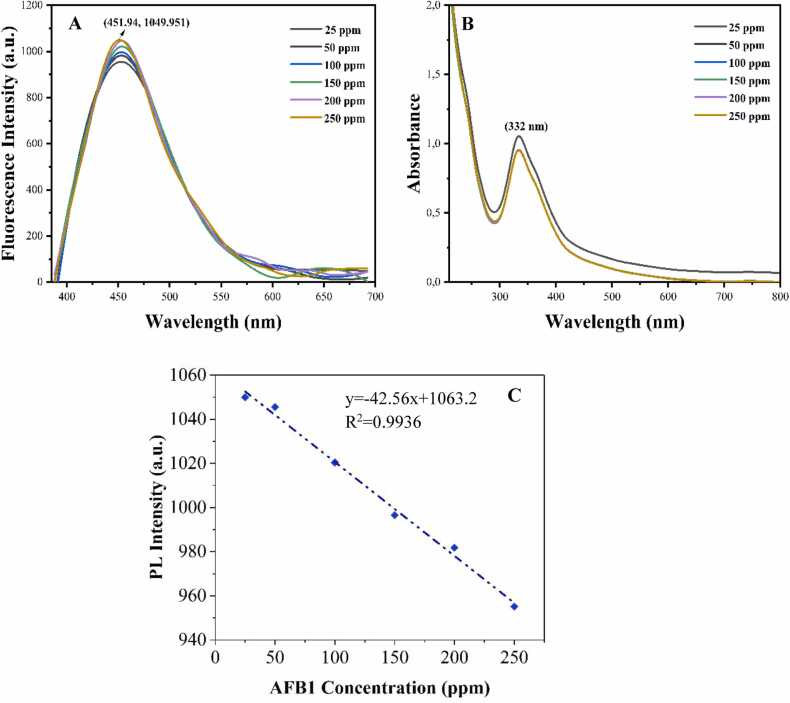
(A) Photoluminescence (PL) spectra (A), absorbance measurements (B) of HH-CQDs in the presence of AFB1 at various concentrations (25–250 ppm) and calibration curve for AFB1 detection based on the fluorescence quenching by HH-CQDs (**C**).

Given that both HH-CQDs and AFB1 exhibit fluorescence, we investigated their mutual effects on each other's fluorescence properties. To evaluate the potential effect of HH-CQDs on AFB1, we recorded the fluorescence spectra of a series of solutions containing AFB1 and HH-CQDs at constant HH-CQD concentrations but with increasing AFB1 concentrations (Fig. 6 A). Our findings show that the presence of HH-CQDs significantly enhances the fluorescence of AFB1 (Fig. 6 A). This enhancement effect highlights the potential of HH-CQDs in the sensitive detection of AFB1. Additionally, UV-Vis spectra of the samples were obtained to further analyze this interaction (Fig. 6B).

The fluorescence enhancement observed suggests that HH-CQDs may interact with AFB1 via mechanisms such as energy transfer or the formation of fluorescent complexes. This interaction enhances the sensitivity of AFB1 detection, which is advantageous for applications in food safety and environmental monitoring. A standard curve was obtained by plotting fluorescence intensities against AFB1 concentrations (Fig. 6 C). Photoluminescence (PL) spectral data indicated that the addition of AFB1 did not alter the emission peak position of HH-CQDs, though the intensity varied significantly between spectra. A strong linear relationship was established between AFB1 concentration and PL peak intensity within the range of 25–250 ppm (R² = 0.9936). The corresponding linear fit equation is y = 37.312x + 222.02.

AFB1 detection using HH-CQDs relies on fluorescence-based mechanisms, primarily fluorescence resonance energy transfer (FRET) and non-covalent interactions. The aromatic structure of aflatoxins facilitates energy transfer from CQDs, leading to changes in their fluorescence intensity. Additionally, functional groups on the CQDs surface, such as –OH, –NH_2_, and –COOH, enable hydrogen bonding, π-π interactions, and van der Waals forces with aflatoxins, enhancing selectivity and sensitivity. These interactions allow for the efficient and sensitive detection of AFB1, making CQDs a promising tool for food safety and environmental monitoring.

In the specificity experiment for AFB1 detection, cysteine, dopamine, vitamin E, ascorbic acid, phenol, and folic acid were added to a solution containing HH-CQDs. AFB1 was set as the reference (100*%*) for fluorescence change, and the relative responses of these other compounds were measured. Cysteine (34.3*%*), dopamine (35*%*), vitamin E (35.6*%*), ascorbic acid (26.3*%)*, phenol (10.7*%*), and folic acid (24*%*) showed significantly lower fluorescence changes compared to AFB1. This significant difference indicates that HH-CQDs have high specificity for AFB1 detection. Although cysteine, dopamine, and vitamin E caused moderate changes, they were well below the response to AFB1, indicating excellent selectivity. Even lower responses of ascorbic acid, phenol, and folic acid further prove the method's robustness, especially in complex environments such as food safety and environmental monitoring.

**Fig. 7:**
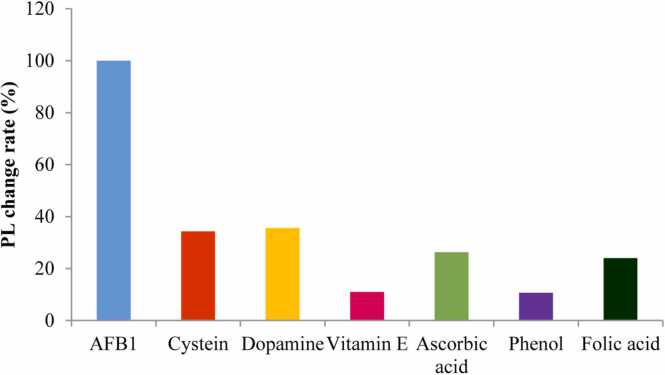
Specificity results of HH-CQD for AFB1 against some chemicals.

AFB1 is thought to lead to enhanced fluorescence signals because of its molecular structure, which allows for more efficient energy transfer between itself and HH-CQDs. The absence of similar interactions with other compounds may lead to lower fluorescence changes. In addition, HH-CQDs can form specific stable complex structures with AFB1 structure through hydrogen bonding and π-π interactions or weak binding such as van der Waals forces due to their carboxyl, amino, and hydroxyl groups. The planar, aromatic structure of AFB1 is of great importance in its selective detection. Similar findings have been reported where non-aromatic compounds exhibited significantly weaker interactions with fluorescent probes due to the lack of effective energy transfer mechanisms [32]. In this way, HH-CQDs provide high selectivity and sensitivity for AFB1 detection, making them a promising tool for food safety and environmental monitoring applications. Fig. 7

**Fig. 8:**
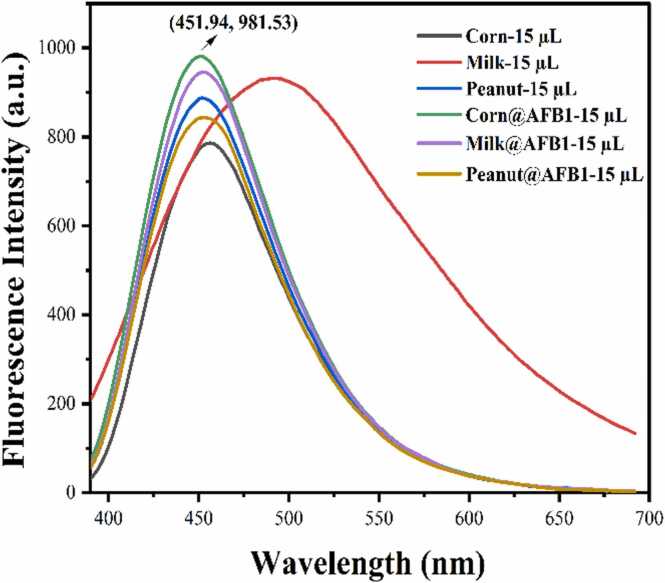
Fluorescence intensity measurements for the determination of the amount of AFB1 in real samples of corn, milk, and peanut.

Fluorescence intensity measurements of the complex obtained by adding HH-CQD to real samples of milk, corn, and peanuts to which AFB1 was added at a concentration of 25 ppm were made, and the results are given in Fig. 8. The amount of AFB1 in real samples was calculated by calculating the obtained PL intensity differences and using the calibration curve graphic equation in Fig. 6 C to calculate the amount of AFB1. Table 1 presents the accuracy rates of the obtained results. The obtained findings revealed high accuracy rates for AFB1 detection, including 82.56*%* in corn, 98.64*%* in milk, and 95.73*%* in peanut.

**Table 1:** Accuracy percentage in the determination of AFB1 in the corn, milk, and peanut samples.

**Food samples+AFB1–25 ppm)**	**Calculated AFB1 concentration (ppm)**	**Percentage accuracy rate**
**Corn+AFB1**	20.39	81.56 %
**Milk+AFB1**	24.66	98.64 %
**Peanut+AFB1**	23.93	95.73 %

## 4 Discussion

In this study, carbon quantum dots (CQDs) obtained from hazelnut shell (FK) waste were synthesized with a rapid and environmentally friendly method, and these dots were used in nanobiosensor applications for the detection of aflatoxin B1 (AFB1). The high solubility, stability and biocompatibility of FK-CQDs against water made them suitable candidates for food safety applications. Among the CQDs synthesis methods previously reported in the literature, the importance of environmentally friendly synthesis methods is increasing. Especially CQDs obtained from agricultural wastes provide great advantages in terms of both sustainability and low cost. In previous studies, CQDs synthesis from various biological wastes such as orange peel waste, corn cob, walnut shell was reported and these gave successful results especially in biosensor and biomedical applications [33], [34], [35].

Structural analysis showed that FK-CQDs were well-dispersed, spherical nanoparticles with an average size of approximately 2.08 nm. Previous studies reported that CQDs obtained from agricultural wastes exhibited similar structural properties. For example, it was reported that CQDs obtained from rice hulls had similar sizes and amorphous structure. Broad peaks were observed in XRD and XPS literature, which were consistent with previously reported CQDs and supported the existence of graphite structure. In XPS analysis, it was also observed in this study that nitrogen-doped CQDs improved optical and electronic properties; this situation increased the fluorescence properties of CQDs and enabled them to be used in biosensors [36], [37].

The fluorescence and UV–vis spectrum results revealed that FK-CQDs showed a strong emission peak at 451 nm at 370 nm excitation wavelength. These optical properties are similar to many biomaterial-based CQDs previously reported in the literature. For example, CQDs obtained from walnut shell exhibited a similar strong fluorescence property and were successful in biosensor applications. Furthermore, the different fluorescence emissions of FK-CQDs as the excitation wavelength was associated with surface defects and different electronic transitions (π-π* and n-π*), which have been similarly explained in the literature previously [35].

The quantum yield of FK-CQDs (4*%*) is in a suitable range compared to other CQDs reported in the literature, highlighting the advantages of low-cost and environmentally friendly synthesis methods. Previously, CQDs obtained from walnut shells were reported to have quantum yields in the range of 5–6*%*, and it was observed that hazelnut shells used in this study provided a similar yield for CQDs synthesis. However, the stability of FK-CQDs and their biosensor application stability make them quite effective in practical applications. It has been emphasized in previous studies that nitrogen doping increases the optical properties of CQDs and their sensitivity in AFB1 detection [38], [39], [40].

The findings obtained from this study revealed that FK-CQDs exhibited pH-sensitive optical properties. Especially in slightly basic environments (pH 9–10), the fluorescence intensity increased and the emission peak shifted to blue. This property is consistent with other previously reported CQDs. For example, it was stated that CQDs obtained from biowaste showed higher fluorescence intensity in basic environment and this advantage could be used in biosensors. Similarly, the decrease in fluorescence in acidic environments and the red shift was explained by the protonation of functional groups, and this situation has been reported in the literature in a similar way [41], [42].

The stability and selectivity of FK-CQDs in the detection of AFB1 were attributed to the presence of functional groups, especially nitrogen doped (–OH, –NH₂ and C=O). Previously reported CQDs-based biosensors in the literature have also yielded successful results in the detection of toxins such as aflatoxin. For example, the success of CQDs obtained from citrus peel in the detection of AFB1 is consistent with the results of FK-CQDs in this study. It has been emphasized in previous studies that nitrogen doping provides CQDs with a stronger interaction against toxins [43], [44].

## 5 Conclusion

Sustainable and eco-friendly approaches to nanomaterial synthesis are gaining significant attention due to their environmental, economic, and societal benefits. In this study, carbon quantum dots (HH-CQDs) were successfully synthesized from hazelnut husk waste using a simple, cost-effective, and eco-friendly pyrolysis method. The use of waste products significantly reduces production costs, contributing to economic efficiency. This approach also offers a sustainable opportunity due to the wide availability of hazelnut production and the valorization of agricultural waste materials. Moreover, it addresses environmental concerns by reducing pollution caused by waste disposal. The synthesized HH-CQDs exhibited strong photoluminescence, excellent stability, high water solubility, and other properties that make them highly suitable for biosensing applications. Characterization techniques such as TEM, FTIR, XRD, and XPS confirmed their spherical morphology, graphitic structure, and functional groups essential for interaction with aflatoxin molecules. The quantum yield of the HH-CQDs was calculated to be 0.04, demonstrating sufficient optical efficiency for biosensing purposes. Furthermore, pH- and temperature-dependent studies indicated the adaptability and sensitivity of HH-CQDs, which could be exploited for the detection of aflatoxin B1 (AFB1) in food samples. Nitrogen- and oxygen-containing functional groups present on the surface of HH-CQDs were identified as key factors in enhancing their interaction with AFB1, providing a sensitive and selective detection platform. These findings suggest that HH-CQDs derived from agricultural waste could play a pivotal role in the development of cost-effective, eco-friendly, and efficient biosensors for food safety monitoring.

## Scientific responsibility statement

The authors declare that they are responsible for the article’s scientific content, including study design, data collection, analysis and interpretation, writing, some of the main lines, or all of the preparation and scientific review of the contents and approval of the final version of the article.

## Animal and human rights statement

All procedures performed in this study were in accordance with the ethical standards of the institutional and/or national research committee and with the 1964 Helsinki Declaration and its later amendments or comparable ethical standards. No animal or human studies were carried out by the authors of this article.

## Funding

This study was supported by Ataturk University, Scientific Research Projects Commission under grant no: FDK-2022–10569. We appreciate Ataturk University's Scientific Research Projects.

## CRediT authorship contribution statement

**Hatice Yuncu:** Writing – review & editing, Writing – original draft, Methodology, Investigation, Conceptualization. **Ebru Bozkurt:** Writing – review & editing, Writing – original draft, Methodology, Data curation, Conceptualization. **Hayrunnisa Nadaroglu:** Writing – review & editing, Writing – original draft, Supervision, Methodology, Investigation, Funding acquisition, Data curation, Conceptualization.

## Declaration of Competing Interest

The authors declare that they have no known competing financial interests or personal relationships that could have appeared to influence the work reported in this paper.

## Data Availability

The authors do not have permission to share data.
